# Negative Effects of Embodiment in a Visuo-Spatial Working Memory Task in Children, Young Adults, and Older Adults

**DOI:** 10.3389/fpsyg.2021.688174

**Published:** 2021-09-13

**Authors:** Gianluca Amico, Sabine Schaefer

**Affiliations:** Department of Sport Sciences, Saarland University, Saarbrücken, Germany

**Keywords:** embodiment, memory, cognition, spatial information, age comparison

## Abstract

Studies examining the effect of embodied cognition have shown that linking one’s body movements to a cognitive task can enhance performance. The current study investigated whether concurrent walking while encoding or recalling spatial information improves working memory performance, and whether 10-year-old children, young adults, or older adults (*M*_*age*_ = 72 years) are affected differently by embodiment. The goal of the Spatial Memory Task was to encode and recall sequences of increasing length by reproducing positions of target fields in the correct order. The nine targets were positioned in a random configuration on a large square carpet (2.5 m × 2.5 m). During encoding and recall, participants either did not move, or they walked into the target fields. In a within-subjects design, all possible combinations of encoding and recall conditions were tested in counterbalanced order. Contrary to our predictions, moving particularly impaired encoding, but also recall. These negative effects were present in all age groups, but older adults’ memory was hampered even more strongly by walking during encoding and recall. Our results indicate that embodiment may not help people to memorize spatial information, but can create a dual-task situation instead.

## Introduction

The human brain can store almost unlimited amounts of information in its episodic memory. Many of these memories contain information about the environment and the position of one’s own body (e.g., when trying to remember where you bought a shirt, information about the store and how you went there are reactivated). Perceiving and encoding multimodal information leads to stronger memories compared to information that was encoded by only one modality (Jahn and Engelkamp, 2003; Feyereisen, 2009). This phenomenon has also been shown in the context of the intersensory-redundancy hypothesis (Bahrick and Lickliter, 2014). This hypothesis states that if the same information is perceived by more than one modality (e.g., seeing a speaker’s mouth while hearing the sound of his/her voice), amodal information like speech rhythm can be perceived more easily, and is more likely to be encoded (intersensory facilitation). Bahrick and Lickliter (2014) emphasize the role that intersensory redundancy plays in the development of selective attention in infancy and early childhood.

Self-performed tasks are multimodal, meaning that multiple sensory systems are involved during encoding (Bäckman et al., 1986). One of these modalities is proprioception, which provides information about one’s own body movements. In our example of purchasing a shirt, this means that you will have a stronger memory of it if you tried it on and felt its material and shape rather than just looked at it. Sensorimotor information can improve memory even more when the respective body movement is meaningfully related to the cognitive processes (Bäckman et al., 1986; Mavilidi et al., 2016; Hainselin et al., 2017; Skulmowski and Rey, 2018). The embodied cognition framework (Barsalou, 2008; Glenberg, 2010) claims that sensorimotor experiences and bodily states are essentially involved in higher cognitive processes. It postulates that the human mind is grounded in mechanisms that have evolved from interaction with the environment, including sensory processing and motor control (Wilson, 2002). According to this framework, sensory processes not only contribute to creating memories, but are also reactivated during recall to simulate past experiences (Barsalou, 1999). These simulations are rich in detail because of their multimodal structure, since they encompass relevant motor and mental states that are part of the original experience (Dijkstra and Zwaan, 2014). The embodied cognition framework has attracted a lot of research attention in the last years (see reviews by Beilock, 2008; Kiefer and Trumpp, 2012; Loeffler et al., 2016; Krüger and Ebersbach, 2018). Studies on the mirror neuron system also support an embodied view on cognition (Hauk and Pulvermüller, 2004; Tettamanti et al., 2005). However, the approach has also been criticized for not being clearly distinguishable from more traditional cognitive accounts (Mahon and Caramazza, 2008; Caramazza et al., 2014).

An interesting aspect of embodiment effects relates to their developmental trajectory: Does embodiment exert similar effects across the lifespan, or are there age-related changes in its influence on cognition? Recent studies indicated that young children may profit more from embodiment than older children or adults do (Link et al., 2013; Hainselin et al., 2017; Schaefer, 2019). This is in line with Piaget’s theory of cognitive development (Piaget, 1975), emphasizing that physical experiences are substantial in the very early stages of the lifespan and become less important with increasing age. Pouw et al. (2014) also expected larger effects of embodiment in children, as learners disembed their mental activity from the environment over time.

In a lifespan approach of embodied cognition, Loeffler et al. (2016) argued that embodied cognition effects are driven by two underlying processes, either by “new” associations (e.g., learning how shape influences the movements of new objects) or by “reactivated” associations (e.g., remembering the flight quality of an American football that you have experienced before). “New” associations imply that the sensorimotor information that is generated during action (e.g., when interacting with an object) supports the generation of conceptual knowledge, while “reactivated” associations refer to how previous experiences influence the encoding of new information (e.g., anticipating the landing location of an American football on the basis of its flight qualities). Loeffler et al. (2016) stated that children may be more dependent on new associations compared to older adults, and are therefore more strongly affected by embodiment effects involving the formation of those associations. On the other hand, older adults may profit more from the reactivation of previously experienced associations, because their accumulated life experiences increase the likelihood of encountering situations that are similar to past experiences. A recent study by Wermelinger et al. (2019) on action perception and action production also adopted a lifespan approach. Older adults were more successful in predicting the consequences of unfamiliar actions compared to younger individuals, which may be related to their accumulation of motor experience with different actions over the lifespan.

While Loeffler et al. (2016) emphasized the benefits that embodiment may have for older adults, Costello and Bloesch (2017) argued that older adults may be generally less embodied than younger adults, because they rely more on visual processing and less on bodily factors (kinesthetic, tactile, proprioceptive). Furthermore, there is a rich literature on older adults’ increased likelihood to show more pronounced performance deteriorations in cognitive-motor dual-task situations (for reviews, see Woollacott and Shumway-Cook, 2002; Al-Yahya et al., 2011; Schaefer, 2014). In part, this may be due to age-related declines in sensory and motor abilities, increasing the need to invest mental resources like attention into the motor domain. According to cognitive load theory (see Sweller et al., 1998, 2019; Brünken et al., 2003), full-body movements that increase cognitive load may reduce the performance of concurrent cognitive tasks, for example by interfering with memory encoding strategies (Amico and Schaefer, 2020). In contrast, the embodied cognition literature would expect meaningful full-body movements to decrease cognitive load, e.g., by “offloading” cognitive content to the environment (Wilson, 2002). The current study investigates embodiment effects over the lifespan, by comparing children to young and older adults, with the aim to disentangle embodiment and dual-task effects.

Recent studies have shown that embodied training can effectively improve cognitive processes of learning and memory in children, adults, and older adults (Dijkstra et al., 2007; Fischer et al., 2011; Link et al., 2013; Moreau et al., 2015). In this context, Kontra et al. (2015) could demonstrate that an embodied science teaching improves memory performance. College students who physically experienced angular momentum by spinning wheels themselves (embodied condition) answered more questions correctly in a later quiz about angular momentum and torque compared to a group of observers who were not exposed to any motor experience themselves. Furthermore, the significant improvement in learning correlated with the activation of sensorimotor brain regions when students reasoned about angular momentum. This relates well to Barsalou’s (1999) concept of embodied simulations, which states that sensory processes are reactivated during recall to simulate the past event. In addition, Lindgren et al. (2016) were able to show the positive effects of embodiment when teaching students aged 12–13 years about gravity and planetary motion. They used an immersive and interactive simulation in which students moved an asteroid using their own body. The control group used a desktop version of the same simulation. The embodied interactive simulation improved the learning gains, engagement, and attitude of students toward science.

In another study, Dijkstra et al. (2007) were able to show that young and older adults can profit from embodiment. Participants had to recall eight memories in combination with either one congruent or one incongruent body posture (an example of a congruent posture would be to lie down while remembering one’s last dental treatment). Both age groups recollected more memories associated with a congruent body posture in a free recall test 2 weeks later. The positive effect of congruent body movements and self-performed tasks over non-movement conditions is also effective when memorizing vocabulary and phrases (see Jahn and Engelkamp, 2003; Manzi and Nigro, 2008; Feyereisen, 2009).

The close relationship between spatial cognition and motor processes has been shown for numerous cognitive tasks including mental rotation (Amorim et al., 2006), spatial visualization (Chu and Kita, 2011), and mental imagery (Frick et al., 2009). Krüger et al. (2014) asked young adults in a mental rotation task whether two simultaneously presented stimuli were identical. The images either showed simple cubes, cubes with colored ends, cubes with body parts in anatomically possible locations, or cubes with body parts in anatomically impossible locations. Participants were fastest and made fewer errors in the possible-body condition, and were slowest and made most errors in the impossible-body condition. The authors concluded that embodiment is an inflexible mechanism that cannot be willingly turned off, such that it may even impede performance under certain circumstances.

Meaningful body movements like gestures may be able to reduce cognitive load and thus improve performance in learning lists of items (Goldin-Meadow et al., 2001), in mental rotation tasks (Ehrlich et al., 2006), and in navigation tasks (So et al., 2014). So et al. (2015) were able to show that gestures are even more effective than spatial language is during rehearsal of spatial information. In their study, young adults learned routes that contained lines with varying directions (vertical, horizontal, diagonal, etc.). In a between-subjects design, participants remembered more routes correctly in conditions in which they were allowed or explicitly encouraged to use gestures during rehearsal as compared to conditions in which they were allowed or encouraged to use spatial language, or conditions in which gestures, language, or both strategies were prohibited.

A recent study by Schaefer (2019) on embodied cognition effects used full-body movements in spatial working memory. She tested 7- and 9-year-old children in a spatial version of a 2-back task and young adults in a spatial 3-back task. Stimuli were presented in a row of nine adjacent fields depicted on the floor. Target fields turned red. The task instruction was to indicate whenever a stimulus was presented at the same position as the stimulus presented *n* positions before (2-back or 3-back, depending on the age group) by saying “tap.” In a within-subjects design, participants either stepped into the target fields while working on the n-back task (embodied condition) or stood still (control condition). The results showed performance improvements in the embodied condition for 7-year-olds, but not for 9-year-olds and young adults. The author argued that the use of more efficient memory strategies in older children and adults may have blurred the beneficial effects of embodiment in the older groups.

Amico and Schaefer (2021) recently attempted to replicate the positive effect of embodiment using a verbal memory task. Children, teenagers, and young adults participated in the study. In a within-subjects design, they were asked to encode strings of numbers, which were either presented while sitting, or with the instruction to move to the corresponding position in space (numbered gymnastic mats). Contrary to predictions, embodiment did not increase recall performances, but led to inferior performances as compared to the sitting condition. Only the youngest age group (8-year-olds) did not show performance differences between the sitting and embodied condition. The authors proposed that the need to move to the respective location, in addition to the interference created by a group setting, may have led to the findings. These assumptions were supported by the second experiment, in which 7-year-olds and young adults were tested in individual test sessions. In this case, there were no costs, but also no benefits in the embodied condition compared to a standing condition. In addition, target numbers were always presented verbally, visually, and as a spatial location, leaving participants with numerous potential strategies to encode the number sequence (i.e., verbal rehearsal or the encoding of spatial positions). A stronger and more exclusive reliance on spatial information ought to increase the chances of finding positive effects of embodiment on spatial memory.

The current study aims to further contribute to the understanding of embodiment in spatial memory, since previous studies could not consistently show positive effects of embodiment. The current literature about embodiment in spatial memory has mainly focused on episodic memory tasks using small-scale movements like gestures. In addition, only very few studies were able to contribute to a lifespan perspective on embodiment. Therefore, the current study investigates possible effects of embodiment in children, young adults, and older adults in a spatial working memory task using full-body movements. Spatial memory develops rapidly from infancy to the preschool years, reaches a plateau in young adulthood, and declines again in older adulthood. At the age of 10 years, children have achieved an advanced hierarchical coding system that continues to develop until young adulthood (Newcombe and Sluzenski, 2004; Newcombe and Learmonth, 2005). Young adults show higher spatial and verbal working memory performance compared to children and older adults (Jenkins et al., 1999). In older adults, starting at the age of around 60 years, spatial memory progressively declines with increasing age (Hedden and Gabrieli, 2004; Colombo et al., 2017; Lester et al., 2017). We tested children at the age of 10 because they were already able to follow the instructions of the tasks, while still differing from young adults in their spatial memory capacity (Zald and Iacono, 1998). Older adults beyond the age of 60 should be affected by age-related declines in cognitive performance.

To measure spatial memory performance in an embodied setting, we decided to use a customized version of a standardized psychological test, namely the Corsi Block-Tapping Task (CBT), a well-known test to measure memory span (Corsi, 1972). The CBT consists of nine small blocks positioned in a standard random configuration on a board. The participant’s task is to memorize and recall sequences of blocks that the test administrator has pointed at. The sequences increase in length over the trials until the participant fails to correctly recall the sequences on several consecutive trials. A study by Piccardi et al. (2008) showed that the CBT can be transferred into a larger room. In the so-called Walking Corsi Task (WalCT), nine target fields are positioned on the floor in the same pattern as in the original test. Instead of using a finger to point at the target fields, the participant and the experimenter step into the fields. Piccardi et al. (2014) showed that the memory performance of children was better in the traditional CBT compared to the WalCT, while the performance of young adults was better in the WalCT compared to the CBT (Piccardi et al., 2008). The authors argued that the CBT tests peripersonal memory (in reaching space), whereas the WalCT measures extrapersonal memory (topographical space). They concluded that extrapersonal span and peripersonal span may change from child- to adulthood. However, differences in inter-stimulus intervals (ISIs) between the CBT and the WalCT may also have caused this result. In the WalCT, the experimenter and the participants take more time to walk to the fields compared to the CBT, where they only have to point at the blocks. In children, this prolonged time between stimulus presentation and recall can lead to performance degradation, as young children lack the ability to use mnemonic strategies (e.g., rehearsal), while young adults may profit from the extra time by using efficient mnemonic strategies (Ornstein, 1978). We therefore argue that it is important to use the same ISIs in embodied and control conditions to reveal potential advantages of embodiment.

In a within-subjects design, the current study tested four different conditions where encoding and recall were carried out while either standing or walking (embodied). Embodiment effects have been shown for cognitive domains like episodic memory (Dijkstra et al., 2007), science learning (Kontra et al., 2015; Lindgren et al., 2016), learning number magnitude representations (Link et al., 2013), mental imagery and rotation (Frick et al., 2009; Krüger et al., 2014), and spatial memory (Rieser et al., 1994; So et al., 2014, 2015; Schaefer, 2019). We therefore predicted that embodied conditions would lead to better memory performance than non-embodied conditions (encoding or recall while walking > encoding or recall while standing). Concerning age-specific outcomes, we expected children to profit more from embodiment than young adults, as indicated by theoretical assumptions (Piaget, 1975; Pouw et al., 2014) and experimental studies (Rieser et al., 1994; Link et al., 2013; Hainselin et al., 2017; Schaefer, 2019). On the other hand, we expected older adults to be impaired by the walking conditions due to their increased need to compensate for sensory and motor declines by investing attentional resources into motor tasks (Woollacott and Shumway-Cook, 2002; Schaefer, 2014), and because older adults may be less embodied than younger participants (Costello and Bloesch, 2017).

Furthermore, it seemed possible that the effect of context-dependent memory would influence memory performance (see meta-analysis by Smith and Vela, 2001). In a classical study, Godden and Baddeley (1975) tested divers in a free recall task. Participants learnt and recalled lists of words either on land or underwater. The results showed that more words could be recalled when learning and retrieval had taken place in the same environment. McClelland and Rumelhart (1985) explained retrieval as a reinstatement of prior patterns of activation, meaning that sensory cues, which are a fragment of the original state, are used to reinstate the mental state experienced during prior activation (see also Dijkstra and Zwaan, 2014). In the current study, this sensory input did change with conditions (recall/encoding while standing or walking), which created context-dependent cues that could affect retrieval. Therefore, we expected better performance in congruent conditions (encoding and recall while walking, henceforth denoted as “walking-walking” and encoding and recall while standing, henceforth denoted as “standing-standing”) compared to incongruent conditions (encoding while walking and recall while standing, “walking-standing” and encoding while standing and recall while walking, “standing-walking”), with the congruent embodied condition (walking-walking) leading to the best memory performance.

The predicted performance patterns were preregistered and can be found using the following links: young adults: https://aspredicted.org/blind.php?x=nm2sc5; children and older adults: https://aspredicted.org/blind.php?x=3bd6vq.

## Materials and Methods

### Participants

Previous embodiment studies revealed large- to medium-sized effects (Manzi and Nigro, 2008; So et al., 2014, 2015). An *a priori* power analysis with power (1-β) set at 0.80 and α = 0.05 indicated a required sample size of 24 participants per group to detect a medium effect with *f* = 0.25 in a repeated measures ANOVA. Due to the Corona pandemic, we only managed to test 16 children, 28 young adults, and 20 older adults. Participants were tested in our laboratory at Saarland University or in a room close to a sports club (see Table 1 for descriptives). All young adults were university students taking part for course credit. Children and older adults were contacted through local sports clubs. All participants had normal or corrected-to-normal vision and hearing and signed informed consent. In the case of children, the form was signed by a legal guardian. As a background variable, perceptual speed was measured with the Digit-Symbol Substitution task. Our participants’ scores were comparable to those of corresponding age groups (Wechsler, 1981). The study was approved by the Ethics Committee of Saarland University.

**TABLE 1:** Descriptives for each age group.

	Children	Young adults	Older adults
*N* (males/females)	16 (0/16)	28 (20/8)	20 (10/10)
Age			
*Mean (SD)*	10.5 (1.37)	22.11 (2.33)	72.5 (4.22)
Digit-symbol substitution task [symbols per second]			
*Mean (SD)*	0.41 (0.10)	1.17 (0.16)	0.44 (0.08)

### Experimental Tasks

#### Spatial Memory Task

**FIGURE 1:**
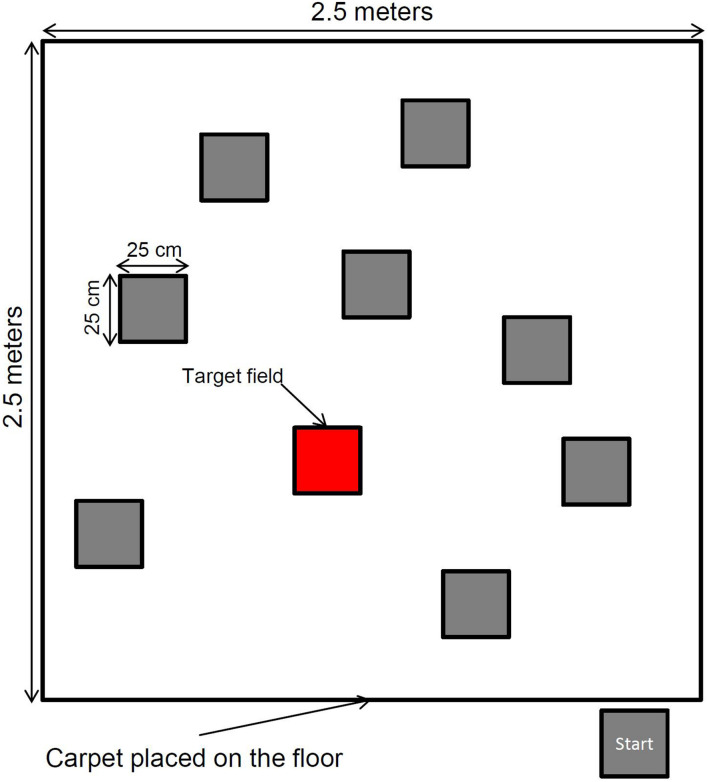
Experimental setup of the spatial memory task, with one illuminated target field.

The Spatial Memory Task is a modified version of the traditional Corsi Block-Tapping Task (CBT) that measures participants’ short-term memory capacity for visuo-spatial information (Corsi, 1972). To investigate the effects of embodiment on visuo-spatial memory, we created a spatial memory task that allows for full-body movement. We positioned 9 target fields (25 cm × 25 cm) in a standard random configuration on a large square carpet (2.5 m × 2.5 m). Participants’ goal in the Spatial Memory Task is to encode and recall sequences of increasing length by reproducing positions of target fields in the correct order. The target fields were illuminated by a beamer that had been mounted to the ceiling. Target stimuli were presented with an ISI of 3 s (see Figure 1 for the experimental setup). Participants encoded the target fields either while standing on the starting field, or while walking to each illuminated field, from target to target. After the last target field of a sequence was presented, a visual signal indicated the end of the trial. The participant then reproduced the series of target fields. Depending on the condition, the series of target fields was either reproduced by pointing at the respective locations with a laser pointer in the correct order, or by walking to the respective target fields. The series lengths ranged from 2 targets to a maximum of 11 targets with 3 trials per sequence length for children and older adults. Young adults started with a sequence length of 3. In total, 4 lists of sequences were created by a computer algorithm. During recall, participants had to indicate the target field for approximately 1 s before pointing or walking to the next target field. The experimenter recorded the sequence of answers for each trial and compared it to a paper-and-pencil template with the correct solution. If a participant failed to reconstruct the sequence correctly in all trials of one difficulty level (e.g., all 3 trials of length 6), the Spatial Memory Task ended. The dependent variable for each condition was the sum of correctly reproduced sequences.^1^ To make the scores of children, young adults, and older adults comparable, the memory performance of the sequences with a length of 3 were scored twice (this compensates for the trials with sequence length 2 that were not carried out in the group of young adults).

### Procedure

Participants were tested individually in two testing sessions. Each session lasted about 1 h. The first started with the Digit-Symbol Substitution task. After that, each participant worked on the Spatial Memory Task under four different conditions: encoding and recall were carried out while either standing or while walking (standing-standing, standing-walking, walking-standing, walking-walking, see above). The order of conditions was counterbalanced with a 2 × 2 Latin Square design, while the lists of stimuli were always used in the same order for each participant (e.g., list 1 for the first trial, list 2 for the second trial, etc.). The encoding condition always stayed the same within one session. Two practice trials were carried out before starting a new condition. The ISI of 3 s was long enough to allow participants to reach the respective target field without running. For exploratory purposes, young adults also performed a tunnel task (Gramann et al., 2005, 2010), which distinguishes the navigational strategies of “Turners” and “Non-Turners,” at the end of the second session. A description of the results of this task and its relation to the present study findings can be found in Supplementary Material 1.

### Data Analysis

The Spatial Memory Task was analyzed with a mixed-design analysis of variance (ANOVA) with condition (4: walking-walking, walking-standing, standing-walking, standing-standing) as the within-subjects factor and age groups (3: children, young adults, older adults) as the between-subjects factor. A second analysis of the Spatial Memory Task was conducted with an ANCOVA to interpret the portion of variance explained by age when controlling for cognitive speed (Digit-Symbol Substitution performance). *F*-values and *partial Eta square* values for effect sizes are reported. The Mauchly-test of sphericity was violated for the within-subjects-factor condition of the ANOVA. Therefore, the respective results are reported using Greenhouse-Geisser corrections. The alpha level used to interpret statistical significance was *p* < 0.05. Significant main effects were further investigated by planned *t*-tests with Bonferroni-corrected levels of significance. For paired-samples *t*-tests, we present Cohen’s *d*_*z*_ effect sizes, and for independent-samples *t*-tests, we present Cohen’s *d* effect sizes.

## Results

**FIGURE 2:**
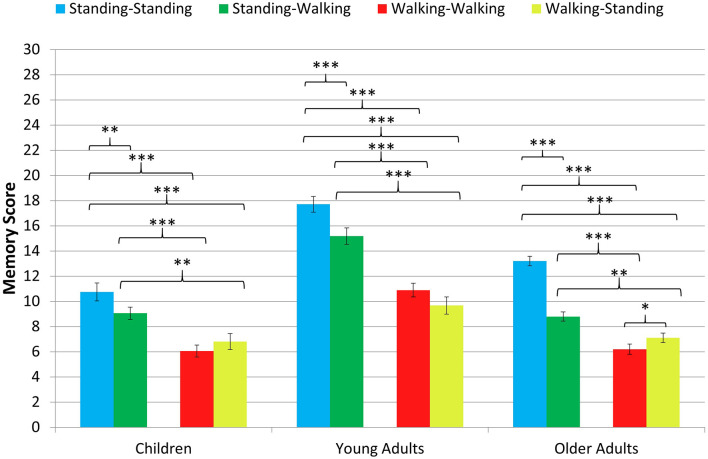
Memory score in the spatial memory task for the four combinations of encoding and recall conditions in each age group. Asterisks indicate the level of significance (*p* < 0.001^∗∗∗^, *p* < 0.01^∗∗^, *p* < 0.05^∗^). Non-significant comparisons are not depicted. Error bars = standard error means.

The results of the ANOVA show a significant main effect of age group, *F*(2, 61) = 48.224, *p* < 0.001, η^2^*_*p*_* = 0.613. Independent *t*-tests with Bonferroni-corrected alpha-error probability to *p* = 0.016 indicate that the young adults’ performance (*M* = 13.37, *SD* = 2.31) was better than that of the children (*M* = 8.17, *SD* = 1.99), *t*(42) = 7.54, *p* < 0.001, *d* = 2.36, and that of the older adults (*M* = 8.83, *SD* = 1.30), *t*(43.95) = 8.67, *p* < 0.001, *d* = 2.54, while children’s performance was comparable to that of the older adults, *t*(34) = 1.19 *p* = 0.242, *d* = 0.40. Figure 2 depicts the pattern of findings for each age group.

**FIGURE 3:**
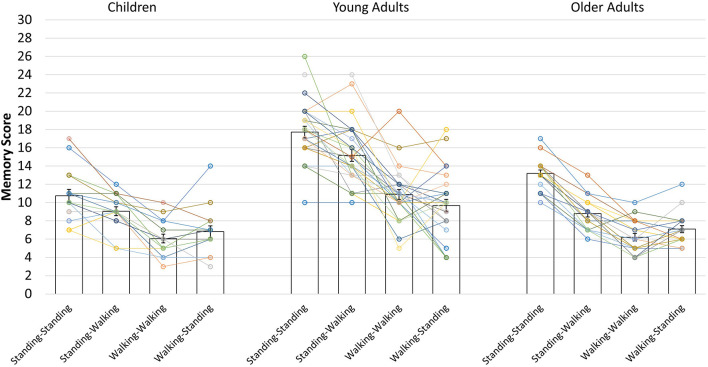
Individual data points of the spatial memory task for the four combinations of encoding and recall conditions in each age group. Bars indicate mean scores with standard error means. Data points connected by lines indicate the performances of single participants in each age group.

Furthermore, the main effect of condition was significant, *F*(2.70, 164.56) = 117.993, *p* < 0.001, η^2^*_*p*_* = 0.659. Paired *t*-tests with the Bonferroni-corrected level of significance to *p* = 0.008 indicated that memory performance was best when participants were standing during encoding and recall (standing-standing condition: *M* = 14.56, *SD* = 4.03), followed by performance in the standing-walking condition (*M* = 11.66, *SD* = 4.08), both of which were significantly better than the walking-walking condition (*M* = 8.22, *SD* = 3.33) and the walking-standing condition (*M* = 8.16, *SD* = 3.14). The difference between the latter two conditions did not reach significance (see Table 2 for follow-up analysis). In addition, there was a significant interaction of condition and age group, *F*(5.40, 164.561) = 5.732, *p* < 0.001, η^2^*_*p*_* = 0.158. Paired-samples *t*-tests with Bonferroni-corrected levels of significance to *p* < 0.008 showed that the pattern of findings is identical to the main effect of condition for children and young adults, but older adults were impaired more strongly by walking during recall. Only older adults showed additional performance decrements in the walking-walking condition compared to the walking-standing condition (see Table 2 for comparisons, Figure 2 for the pattern of findings, and Figure 3 for individual performances by condition).

**TABLE 2:** Follow-up analysis for main effect of condition and the interaction of condition and age group.

	Paired *t*-tests for the main effect condition		Paired *t*-tests for the interaction of condition × age group	
	
Pairs	Overall	Children	Young adults	Older adults
Walk-walk vs. Walk-stand	*t*(63) = 0.16, *p* = 0.873, *d_*z*_* = 0.02	*t*(15) = 1.49, *p* = 0.158, *d_*z*_* = 0.37	*t*(27) = 1.61, *p* = 0.11, *d_*z*_* = 0.30	*t*(19) = 2.39, *p* = 0.027, *d_*z*_* = 0.53
Walk-walk vs. Stand-walk	*t*(63) = 9.03, *p* < 0.001, *d_*z*_* = 1.13	*t*(15) = 6.57, *p* < 0.001, *d_*z*_* = 1.64	*t*(27) = 5.66, *p* < 0.001, *d_*z*_* = 1.07	*t*(19) = 6.40, *p* < 0.001, *d_*z*_* = 1.43
Walk-walk vs. Stand-stand	*t*(63) = 15.07, *p* < 0.001, *d_*z*_* = 1.88	*t*(15) = 8.84, *p* < 0.001, *d_*z*_* = 2.21	*t*(27) = 8.42, *p* < 0.001, *d_*z*_* = 1.60	*t*(19) = 14.23, *p* < 0.001, *d_*z*_* = 3.18
Walk-stand vs. Stand-walk	*t*(63) = 8.74, *p* < 0.001, *d_*z*_* = 1.09	*t*(15) = 4.32, *p* < 0.008, *d_*z*_* = 1.08	*t*(27) = 8.60, *p* < 0.001, *d_*z*_* = 1.63	*t*(19) = 3.96, *p* < 0.008, *d_*z*_* = 0.89
Walk-stand vs. Stand-stand	*t*(63) = 13.88, *p* < 0.001, *d_*z*_* = 1.74	*t*(15) = 7.24, *p* < 0.001, *d_*z*_* = 1.81	*t*(27) = 9.48, *p* < 0.001, *d_*z*_* = 1.79	*t*(19) = 14.03, *p* < 0.001, *d_*z*_* = 3.14
Stand-walk vs. Stand-stand	*t*(63) = 8.78, *p* < 0.001, *d_*z*_* = 1.10	*t*(15) = 3.18, *p* < 0.008, *d_*z*_* = 0.80	*t*(27) = 4.24, *p* < 0.001, *d_*z*_* = 0.80	*t*(19) = 15.46, *p* < 0.001, *d_*z*_* = 3.46

*^1^An alternative analysis of the results using memory span as the dependent variable for memory performance can be found in Supplementary Material 2.*

To investigate how the observed findings are influenced by age group differences in cognition, we ran an ANCOVA controlling for Digit-Symbol scores. The ANCOVA did not reveal a significant main effect of age group, *F*(2, 60) = 0.262, *p* = 0.770, η^2^*_*p*_* = 0.009, but a significant main effect of the Digit-Symbol score, *F*(1, 60) = 12.149, *p* < 001, η^2^*_*p*_* = 0.168, showing that a substantial portion of variance of age group is explained by differences in cognitive speed. However, the interaction of condition and age group continues to reach significance, *F*(6, 180) = 2.841, *p* = 0.011, η^2^*_*p*_* = 0.087, indicating that age group has an effect on performance in the different conditions after controlling for cognitive speed. The interaction of condition and Digit-Symbol score did not reach significance, *F*(3, 180) = 1.218, *p* = 0.304, η^2^*_*p*_* = 0.020.

## Discussion

The current study aimed to provide evidence for the positive effects of embodiment on spatial memory using an extended version of the traditional CBT. The results showed that embodiment (walking during encoding or recall) did not enhance memory performance, but reduced memory performance significantly, especially if participants walked while encoding. Furthermore, we did not find a consistent positive effect of congruent conditions compared to incongruent conditions. Although participants reached the highest scores in the standing-standing condition, their performance levels in the walking-walking condition were very low. This indicates that this result was caused by the negative effect of walking as such rather than by the congruency effect.

Pouw et al. (2014) explained two mechanisms at work behind the embodied cognition framework. The first is “embodiment,” meaning that cognitive processes can draw on previous sensorimotor experiences. The second is “embeddedness,” which states that perceptual and interactive richness may assist in alleviating cognitive load. The current study showed that neither “embodiment” (e.g., conditions where recall took place after encoding with concurrent walking) nor “embeddedness” (e.g., conditions where encoding or recall took place with concurrent walking) enhanced cognition.

Contrary to our predictions, walking to the target fields during encoding and recall led to performance decrements. What mechanisms can explain this finding? Our hypothesis was grounded on the concept of embodied cognition, which states that multi-sensory information, including motor information, can improve memory performance (Barsalou, 1999; Wilson, 2002). In this context, previously experienced sensory information can work as a cue during retrieval, by aiding reinstatement of the same mental state as during encoding (McClelland and Rumelhart, 1985; Dijkstra and Zwaan, 2014). In addition, enhanced memory encoding can be expected when information is perceived by more than one modality during encoding (Bahrick and Lickliter, 2014). However, it is possible that the perception of one’s own motor information interfered with the encoding and recall of the spatial positions. Moving and navigating to the target fields requires constant updates of one’s own position and the positions of the target fields, which possibly created a cognitive-motor dual-task (Kahneman, 1973, 2011; Navon and Gopher, 1979; Schaefer, 2014). Walking while working on an episodic memory task has often been shown to reduce memory performance (Lindenberger et al., 2000; Li et al., 2001; Krampe et al., 2011). In the current study we found that memory performance was particularly disturbed when participants moved during encoding. We assume that encoding the sequence of target fields required more attentional resources than recalling them, leading to more pronounced performance reductions for encoding-while-walking conditions (Wickens, 1980, 1991). Furthermore, the perceived motor information while encoding could have been too undifferentiated to provide helpful cues for the reconstruction of the order of target fields. Instead, it may have interfered with the encoding of other more relevant information (e.g., visual patterns of the fields).

It is possible that our choice of ISIs of 3 s increased task difficulty in the encoding-while-walking conditions. Although participants were always able to reach each target field in time, detecting the next target field, and then walking to it probably required attention, and did not leave room for the use of elaborate encoding strategies. Using identical ISIs for both encoding conditions is an important feature of our experimental paradigm, because allowing for more encoding time in embodied conditions (Piccardi et al., 2008) would not represent fair comparison. Using considerably longer ISIs would enable participants to use more elaborate encoding strategies, for example by creating a spatial representation of the path between individual fields, or by inventing a numeric system to encode individual fields and their order. We assume that increasing the ISIs of task would increase its reliance on episodic memory. Future research should investigate whether permitting longer encoding times between individual stimuli would influence the pattern of results.

Regarding the different age groups, we found comparable performances for children and older adults in the Spatial Memory Task, with young adults showing superior performances. As discussed before, there was no positive effect of embodiment in any of the age groups. In addition, the pattern of results did not differ between children and young adults, forcing us to dismiss our initial hypothesis that children may profit more from embodiment than adults. Although embodiment may help in cognitive tasks that involve learning or understanding (Link et al., 2013; Kontra et al., 2015; Lindgren et al., 2016) and episodic memory (Dijkstra et al., 2007; So et al., 2015), embodiment may be of limited use for enhancing spatial working memory. A recent study by Amico and Schaefer (2021) also failed to find performance enhancements due to embodiment when children, adolescents and young adults were asked to encode number sequences. Embodiment was implemented there by asking participants to move to specific positions in space during encoding. The spatial locations corresponded to the to-be-encoded number. Contrary to predictions, recall performances were decreased when the encoding phase consisted of running to the corresponding gymnastic mat in a gym hall, as compared to a sitting encoding condition, except for the youngest age group (8-year-olds). However, a study by Schaefer (2019) showed positive effects of embodiment in a spatial version of the n-back task in children aged 7 years, but not in 9-year-olds. In the current study, children were about 10 years old, which leaves the possibility that younger children could have profited from embodiment. This assumption can be supported by the developmental interrelatedness of spatial navigation and self-locomotion (Anderson et al., 2013) and by theories of cognitive development that assume that physical experiences are substantial at the very early stages of life and become less important with increasing age (Piaget, 1975). If learners do indeed disembed their mental activity from the environment over time (Pouw et al., 2014), a crucial challenge for future studies is to target suitable age groups to reveal these effects.

We expected older adult’s memory performance to be impaired by the walking conditions due to age-related deteriorations in sensory and motor performances (Woollacott and Shumway-Cook, 2002; Schaefer, 2014). This hypothesis was also supported by the assumption of Costello and Bloesch (2017), who describe older adults as less embodied, because they rely more on visual than on sensorimotor information compared to young adults. In the current study, memory performance was impaired, not only in older adults but also in children and young adults, if they walked during encoding or recall. However, older adults’ performances deteriorated even more strongly compared to the other age groups. We assume that older adults’ limited cognitive resources and their higher need to invest cognitive resources into the motor domain caused these differences. Unlike children and young adults, older adults were less able to compensate for the additional cognitive load of walking during recall. Since gender was not distributed equally across the age groups, we were not able to investigate gender as an additional between-subjects factor. This should be done in further experiments. In addition, future research with larger sample sizes per group should also assess the influence of underlying motor and cognitive skills in each age groups, since the performances in embodied cognition tasks are not only influenced by age *per se*. This is also reflected in the rather large proportion of variance in navigation performance that was explained by the cognitive speed measure (Digit-Symbol score) in the current study.

Finally, we expected equal encoding and recall conditions (standing-standing, walking-walking) to show higher memory performance compared to unequal conditions (standing-walking, walking-standing). In previous studies, information was recalled better if the conditions during recall and encoding stayed the same (e.g., occurring in the same environment, see Godden and Baddeley, 1975; Smith and Vela, 2001). The results of the current study cannot fully support this hypothesis. As shown by the main effect of condition, standing during encoding and recall elicited the highest scores, while walking during encoding and recall resulted in very poor performances. We argue that the negative effects of walking caused these effects, and not congruency. Future studies investigating the effect of context-dependent memory should manipulate the environment or the mental or bodily state of the participants.

It is an open question how embodiment would have affected spatial working memory performance if we had only asked for gestures rather than full-body movements, for example by using the setup of the traditional CBT. Would pointing to or touching the targets during encoding and recall create enough sensory input to be helpful in distinguishing the positions of the targets? A study by Chum et al. (2007) supports this idea. They showed that young adults were better at encoding and retrieving the positions of sequences of circles or squares when they used their finger to point at the targets, compared to their performance when they only watched and verbally encoded the positions. Eliminating full-body movements would also reduce the problem of altered points of view of the respective participant between embodied and non-embodied conditions. These may have increased cognitive load and had consequences for egocentric or allocentric strategies applied in spatial tasks (Chum et al., 2007). In the present context, this could be further elaborated by including a standing condition where participants watch the stimuli while standing in the middle of the carpet rather than standing at the corner of the carpet. It is also possible that the use of full-body movements to encode locations does not occur frequently in our daily life, and thus our participants were not familiar with this strategy. Future studies should familiarize and train participants in the encoding-while-walking condition to investigate whether the negative effects of walking are a result of insufficient training, or whether walking as a memorization strategy generally requires more resources than pointing or watching do. If the latter held, a differentiation between embodiment with and without full-body movements is required, especially with regard to spatial memory.

## Conclusion

In conclusion, further research should determine the mechanisms that underlie the effects of embodied cognition, as it is strongly affected by age, cognitive resources, the kind of movement used, and the type of cognitive task applied. To date, there is a lack of age-comparative studies, and studies investigating embodiment in the domain of spatial working memory. Our findings indicate that embodiment has its limits in improving cognitive performance, and may sometimes even lead to performance deterioration caused by the need for additional body movements.

## Data Availability Statement

The raw data that support the conclusions of this article will be made available by the authors upon request without undue reservation.

## Ethics Statement

The studies involving human participants were reviewed and approved by the Ethics committee of Saarland University. Written informed consent to participate in this study was provided by the participants’ legal guardian/next of kin.

## Author Contributions

GA analyzed and interpreted the data, with input from SS. GA led the drafting of the manuscript, with contributions from SS. Both authors contributed to the study design and cooperated in conducting the literature review.

## Conflict of Interest

The authors declare that the research was conducted in the absence of any commercial or financial relationships that could be construed as a potential conflict of interest.

## Publisher’s Note

All claims expressed in this article are solely those of the authors and do not necessarily represent those of their affiliated organizations, or those of the publisher, the editors and the reviewers. Any product that may be evaluated in this article, or claim that may be made by its manufacturer, is not guaranteed or endorsed by the publisher.
