# Supplementary material for: Negative Effects of Embodiment in a Visuo-Spatial Working Memory Task in Children, Young Adults, and Older Adults
Source: Front Psychol. 2021 Sep 13;12:688174. doi: 10.3389/fpsyg.2021.688174 (PMC8473613; doi:10.3389/fpsyg.2021.688174)
Supplement: Supplementary file 1 [file Data_Sheet_1.pdf]

Supplementary Material: The Influence of Navigational Types

In the WalCT, the participant’s point of view continuously changes while navigating between the target fields. This requires a constant updating of one's own position in relation to the positions of the target fields. Research findings by Gramann and colleagues (2005) indicate that there are two navigational types, called Turner and Non-turner (see also Gramann et al., 2010). Their model assumes that approximately 50% of people prefer to imagine themselves in an egocentric perspective as they navigate mentally through a fictional environment (Turner), whereas the other 50% prefer an allocentric perspective as if they were looking at a map (Non-turner).

It is possible that the navigation type modulates the performance in our Spatial Memory Task. In our experiment, participants either stood still or walked to the target fields while encoding and recalling sequences of target fields on the floor. When standing, the individual participant’s perspective on the target fields stayed the same, but when walking, he/she needs to update his/her location constantly to the new perspective. According to Barsalou’s (2008) theory of grounded cognition, participants may simulate how they walk to the target fields during retrieval. Perhaps they use different perspectives during this simulation depending on their navigation type (Gramann et al., 2005). Turners may profit more from walking into the fields, due to their egocentric navigational strategy, while Non-turners may profit from the stationary view that allows them to use their allocentric navigational strategy. If so, participants identified as Turners should profit more from embodied encoding and recall conditions than Non-turners. We therefore included a measure to identify our young adult participants in the current study as Turners or Non-turners for exploratory purposes.

Method

Tunnel task. The tunnel task is a virtual navigation task that distinguishes between two types of navigational strategies: Non-turners and Turners (Gramann et al., 2005). Young adults watched two short movies that provided visual information of translational and rotational changes, creating a simulation of walking through a tunnel with either one right or one left turn (see Figure 1 for examples). After the young adults watched the respective movie, they were asked to choose between two 3D-arrows, one pointing to the back left, and the other to the back right. They were instructed to choose the arrow pointing back to the virtual position where they think that they entered the tunnel. For the example with the right turn, this would mean that participants who updated their imagined direction (Turners) would choose the 3D-arrow pointing to the back right. Non-turners who did not update their cognitive heading would instead choose the 3D-arrow pointing to the back left in the right turn example. Left turns elicit the opposite pattern of responses. If a participant answered coherently after both movies, he was accordingly labeled as a Turner or Non-turner. If the answers were inconclusive, the participant was labeled as such.

Figure 1.

Example of the Tunnel task showing a right turn

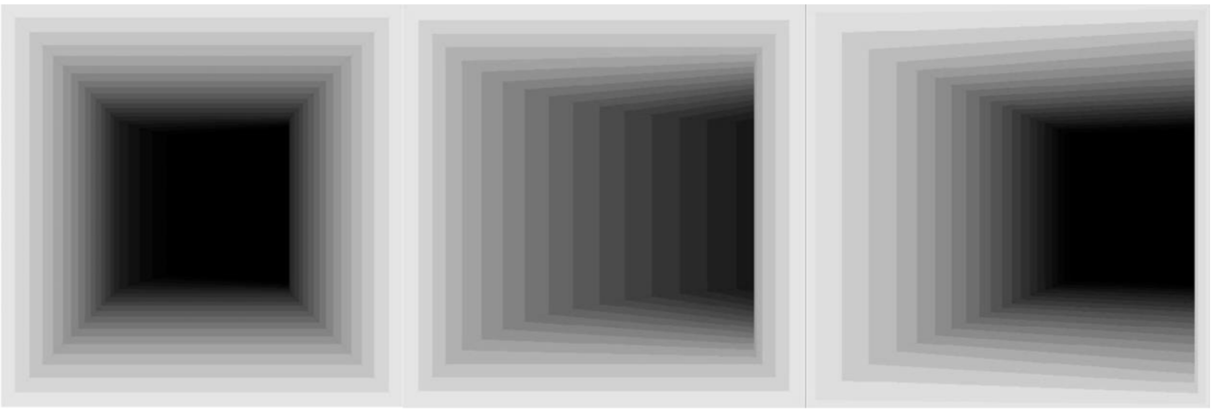

Results

For exploratory purposes, we tested all young adults in the Tunnel task. 15 participants were classified as Turners and 13 participants were classified as Non-turners.

Note that none of the young adults could not be classified consistently. The proportion of Turners and Non-turners in our sample is comparable to the proportions reported by Gramann and colleagues (2005, 2010). To investigate if being classified as a Turner or Non-turner has an influence on the performance in the Spatial Memory Task and affects the encoding and recall condition, we conducted an ANOVA with Condition (4: walking-walking, walking-standing, standing-walking, standing-standing) as the within-subjects factor and Turner/Non-turner as the between-subjects factor.

The ANOVA neither showed a significant main effect of Turner/Non-turner, F (1,26) = .03, p = .859, η^2_p = .001, nor a significant interaction of condition x Turner/Non-turner, F (3,78) = .534, p = .660, η^2_p = .02.

Discussion

Being a Turner (preferring an egocentric heading direction) or a Non-turner (preferring an allocentric heading direction when one imagines navigating through an environment) did not have an effect on memory performance in the current study, independent of encoding or recall condition. We had assumed that Turners might profit from embodied conditions during encoding or recall, whereas Non-turners should show superior performances when encoding or recall are performed without any body movement. Distinguishing between the two types of strategies using the tunnel task worked well, and resulted in percentages of Turners and Non-turners in our young adult sample that corresponded well to previous studies (Gramann et al., 2005, 2010). It is possible that our sample sizes were too small to detect an interaction of navigational strategy and encoding or recall condition. Our study failed to find the predicted positive main effect of embodiment on spatial memory performance in the first place. The detrimental effect of moving to the to-be-encoded (or to-be-recalled) target fields was rather strong. Additional influences of specific preferences for certain navigational strategies (like being a Turner or a Non-turner) are

probably weaker. Future research should test such interactions in larger samples across the lifespan.
