# Supplementary material for: Negative Effects of Embodiment in a Visuo-Spatial Working Memory Task in Children, Young Adults, and Older Adults
Source: Front Psychol. 2021 Sep 13;12:688174. doi: 10.3389/fpsyg.2021.688174 (PMC8473613; doi:10.3389/fpsyg.2021.688174)
Supplement: Supplementary file 2 [file Data_Sheet_2.pdf]

Supplementary Material 2:

Alternative Analysis with Memory Span as the Dependent Variable of the ANOVA

Data analysis

The following analysis uses the Memory Span as the dependent variable of the Spatial Memory Task. The Memory Span score represents the longest sequence length a participant was able to reproduce correctly (i.e., a participant who was able to reproduce at least one trial of a sequence with 7 targets was assigned a span of 7).

The Spatial Memory Task was analyzed with a mixed-design analyses of variance (ANOVA) with condition (4: walking-walking, walking-standing, standing-walking, standing-standing) as the within-subjects factor and age groups (3: children, young adults, older adults) as the between-subjects factor. We report *F* values and *partial Eta square* values for effect sizes. The alpha level used to interpret statistical significance was $p < .05$. Significant main effects were further investigated by planned *t*-tests with Bonferroni-corrected levels of significance. For paired-samples *t*-tests, we present Cohen's d_z effect sizes and for independent samples *t*-tests, we present Cohen's *d* effect sizes.

Results

The results show a significant main effect of age group, F (2,61) = 50.467, $p < .001$, $\eta^2_p = .623$. Independent t-tests with Bonferroni-corrected alpha-error probability to $p = .016$ indicate that young adults' performance (M = 6.66, SD = 1.00) was better than children`s (M = 4.41, SD = 0.86), t (42) = 7.51, $p < .001$, d = 2.35, and older adults' performance (M = 4.65, SD = 0.49), t (41.37) = 9.16, $p < .001$, d = 2.41, while children's was comparable to older adults' performance, t (22.57) = 1.01, p = .323, d = 0.36.

Furthermore, the main effect of condition was significant, F (3,183) = 65.031, p < .001, η^2_p = .516. Paired t-tests with level of significance Bonferroni-corrected to p = .008 indicate that memory performance was best when participants were standing during encoding and recall (standing-standing condition: M = 6.77, SD = 1.72), followed by the standing-walking condition (M = 5.88, SD = 1.83), which were both significantly better than the walking-walking condition (M = 4.56, SD = 1.41) and the walking-standing condition (M = 4.67, SD = 1.41). The difference between the latter two conditions did not reach significance (see Table 2 for follow-up analysis). In addition, there was a significant interaction of condition and age group, F (6,183) = 3.758, p = .001, η^2_p = .110. Paired-samples t-tests with levels of significance Bonferroni-corrected to p < .008 show that older adults' performance was more strongly impaired by walking during recall, while children and young adults were not negatively affected by walking during recall. Only older adults showed additional performance decrements in the standing-walking condition compared to the standing-standing condition. In addition, only older adults showed no significant difference between the standing-walking and the walking-standing condition (see Table S1 for comparisons and Figure S1 for the pattern of findings).

Table S1. *Follow-up Analysis for Main Effect of Condition and the Interaction of Condition and Age Group*

	Paired t-Tests for the Main Effect Condition	Paired t-Tests for the Interaction of Condition x Age group		
Pairs	Overall	Children	Young Adults	Older Adults
Walk-Walk vs Walk-Stand	t (63) = 0.60, p = .548, d_z = 0.08	t (15) = 1.60, p = .158, d_z = 0.40	t (27) = 0.96, p = .344, d_z = 0.18	t (19) = 1.92, p = .070, d_z = 0.43
Walk-Walk vs Stand-Walk	t (63) = 7.68, p < .001, d_z = 0.96	t (15) = 7.25, p < .001, d_z = 1.81	t (27) = 4.92, p < .001, d_z = 0.93	t (19) = 4.41, p < .001, d_z = 0.99
Walk-Walk vs Stand-Stand	t (63) = 12.97, p < .001, d_z = 1.62	t (15) = 7.00, p < .001, d_z = 1.75	t (27) = 7.43, p < .001, d_z = 1.40	t (19) = 11.05, p < .001, d_z = 2.47
Walk-Stand vs Stand-Walk	t (63) = 6.91, p < .001, d_z = 0.86	t (15) = 3.50, p < .008, d_z = 0.88	t (27) = 7.10, p < .001, d_z = 1.34	t (19) = 2.02, p = .058, d_z = 0.45
Walk-Stand vs Stand-Stand	t (63) = 10.40, p < .001, d_z = 1.30	t (15) = 6.62, p < .001, d_z = 1.66	t (27) = 7.17, p < .001, d_z = 1.36	t (19) = 8.01, p < .001, d_z = 1.79
Stand-Walk vs Stand-Stand	t (63) = 5.34, p < .001, d_z = 0.67	t (15) = 2.52, p = .023, d_z = 0.63	t (27) = 2.26, p = .032, d_z = 0.43	t (19) = 8.10, p < .001, d_z = 1.81

Note. The level of significance was Bonferroni-corrected to p < .016.

Figure S1. *Memory Span in the Spatial Memory Task for the Four Combinations of Encoding and Recall Conditions in Each Age Group*

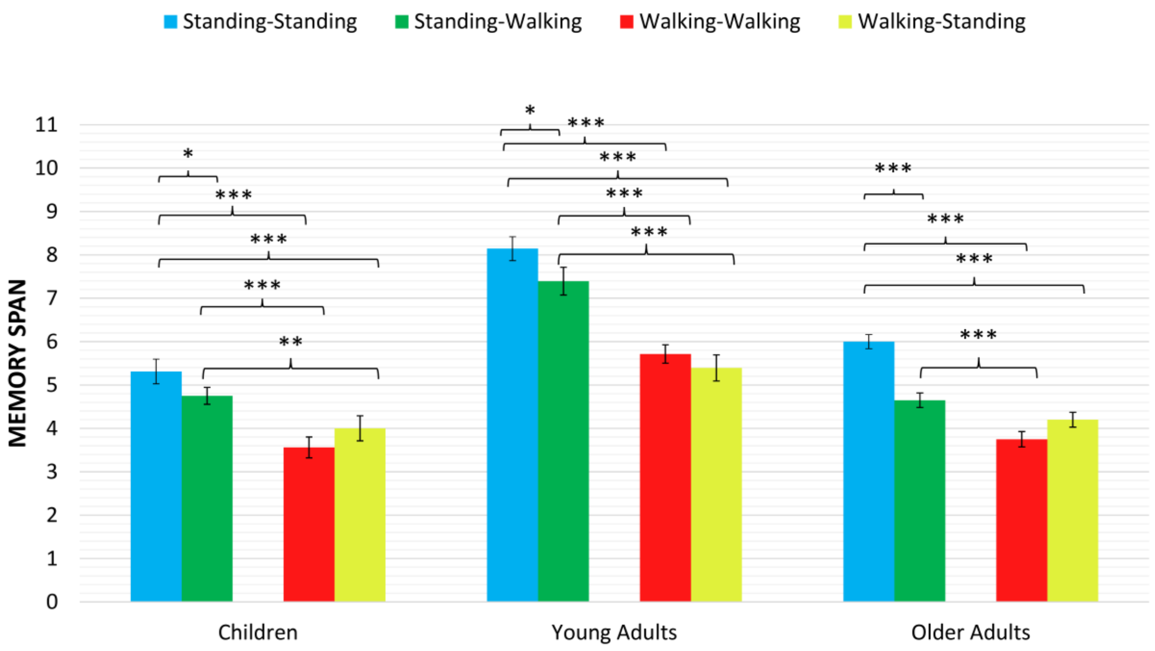

Note. Asterisks indicate the level of significance ($p < .001^{***}$, $p < .01^{**}$, $p < .05^{*}$). Non-significant comparisons are not depicted. Error bars = standard error means.

Discussion

The pattern of results based on the span scores is very similar to the pattern obtained in analyses using the cumulative score as the dependent variable. The only differences are in the paired-samples *t*-tests conducted for the interaction of condition and age group. In the current analysis, the comparison of the standing-standing and the standing-walking condition did not reach significance in children and young adults (with Bonferroni correction), while older adults performed worse in the standing-walking condition. In addition, only older adults showed no difference in performance when comparing the walking-standing and the standing-walking conditions. These results show that the older adults were additionally impaired when walking during recall, while all age groups’ performance decreased when walking during encoding. This interpretation corresponds to the interpretations of the manuscript.
